# Serum troponin I as an indicator of myocarditis in lambs affected with foot and mouth disease

**Published:** 2013

**Authors:** Mohammad Reza Aslani, Mehrdad Mohri, Ahmad Reza Movassaghi

**Affiliations:** 1 *Department of Clinical Sciences, Faculty of Veterinary Medicine, Shahrekord University, Shahrekord, Iran; *; 2 *Department of Clinical Sciences, School of Veterinary Medicine, Ferdowsi University of Mashhad, Mashhad, Iran; *; 3 *Department of Pathobiology, School of Veterinary Medicine, Ferdowsi University of Mashhad, Mashhad, Iran.*

**Keywords:** Foot and mouth disease, Lambs, Myocarditis, Serum troponin I

## Abstract

Foot and mouth disease (FMD) is a highly contagious viral disease of cloven-hooved livestock and wildlife results to relatively high mortality in young animals. Despite the numerous reports of FMD-related death in neonates, there is little data available on various aspects of FMD in lambs. This report describes myocarditis associated with FMD in five, one week to three months old lambs. The lambs were depressed and afebrile and two of lambs showed foamy salivation associated with shallow ulcers in oral cavity. Electrocardiography (ECG) revealed sinus tachycardia, multifocal ventricular premature beats and ventricular fibrillation. Serum biochemistry showed high levels of troponin I concentration and CK and AST activity. In Pathology, there were multiple pale areas in the subepicardial and subendocardial muscles and; widespread degeneration and coagulative necrosis of myocardium. The serum troponin I assay and ECG can be used for diagnosis of myocarditis and prognosis of affected lambs during FMD outbreak.

## Introduction

Foot and mouth disease (FMD) is a highly contagious viral disease of cloven-hoofed livestock and wildlife characterized by the fever and formation of vesicles in the mouth, on the feet, mammary gland and teats in adults. Because of high spread of transmission and great economic consequences the disease is considered as a plague for animal farming.^1^^,^^2^

FMD virus is spread rapidly by airborne route, movement of infected animals and their products; and by mechanical transmission such as people, fomites and vehicles.^3^^,^^4^ FMD is enzootic in large areas of Africa, Asia and South America and has shown an extraordinary ability to cross international boundaries and cause epidemics and losses in previously free areas.^5^ In general, sheep and goats are less susceptible than cattle and pigs and because of mild clinical signs, sometimes it is very difficult to make a clinical diagnosis of FMD in sheep.^6^

In spite of high morbidity and severity of FMD, the case fatality rate is generally low and less than 2% in adults.^2^ However, the mortality in young animals, particularly in lambs and piglets is considerable due to acute myocarditis, the fatal form of FMD without classic vesicular lesions.^5^^,^^7^ Despite the numerous reports of FMD-related death in neonates, there is little data available on various aspects of FMD in lambs. Herein, cardiac biomarkers, electrocardiographic and pathologic features of fatal FMD in lambs are reported which would help to understand the course of the disease and reasons for this high mortality. 

## Case description

**Fig. 1:**
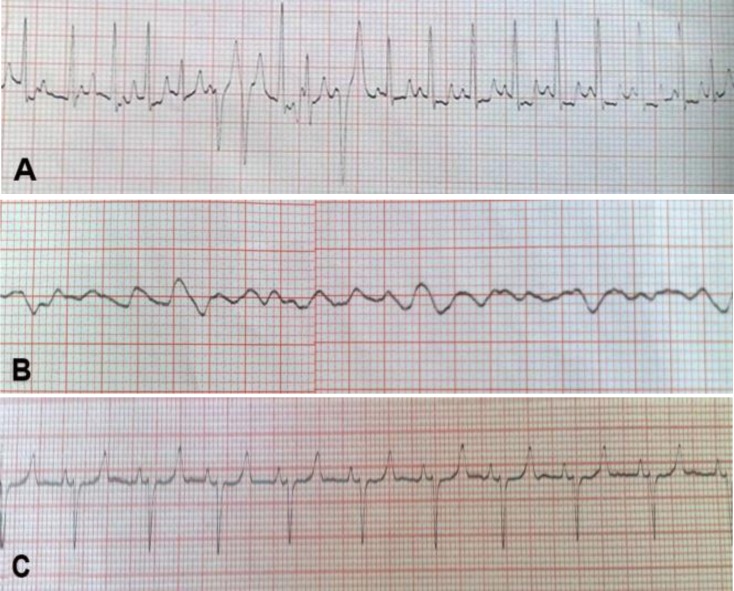
Electrocardiograms of a lamb affected with FMD showing tachy-arrhythmia accompanied by multifocal ventricular premature beats **(A)** and ventricular fibrillation **(B)** 15 minutes later. Strip **(C)** is showing normal ECG of a lamb Base-apex lead, speed 25 mm per sec

The study involved five one week to three months old lambs from three sheep flocks affected with FMD in April and May 2011. Cases were brought to the Teaching Clinic of School of Veterinary Medicine, Ferdowsi University of Mashhad (Mashhad, Iran). The sheep flocks suffered from lamb mortality (25-35%) and FMDV type O was confirmed as a causative agent by local veterinary organization using reverse transcriptase polymerase chain reaction (RT-PCR). The lambs were depressed and afebrile with rectal temperature range of 38.5 to 39.6 ˚C. Two lambs showed foamy salivation associated with shallow ulcers in oral cavity. Tachycardia was noticed in all lambs. Electrocardiograms (ECGs) were recorded using a base-apex lead.^2^ ECGs were evaluated for heart rate and rhythm and abnormalities of conduction. Electrocardiograms showed sinus tachycardia, multifocal ventricular premature beats and ventricular fibrillation (Fig. 1).

**Fig. 2:**
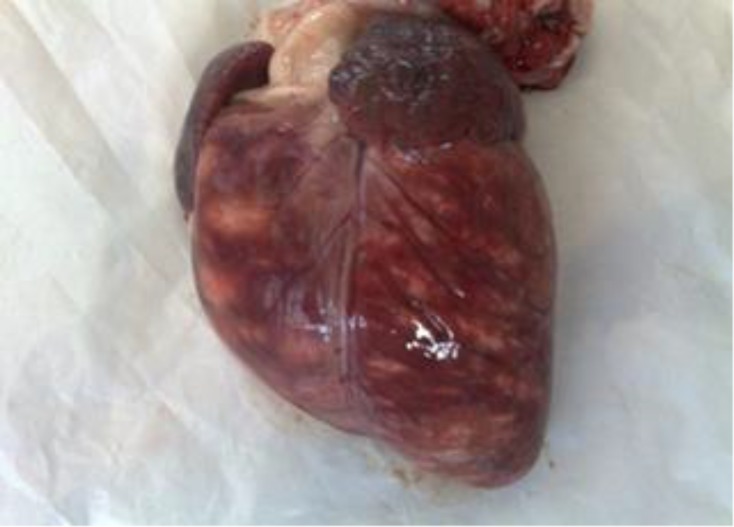
Widespread subepicardial pale areas of necrosis in a lamb with FMD.

Venous blood samples were collected for measurement of serum creatine kinase (CK), aspartate amino-transferase (AST) by an autoanalyzer (Model Targa 3000, Biotechnica instrument, Rome, Italy) using commercial kits (Pars Azmoon, Tehran, Iran). Serum troponin I concentration was determined by an enzyme –linked immunoabsorbent assay (ELISA) kit (Monobind Inc, Lake Forest, CA, USA). The serum toponin I concentration ranged from 2.40 to 30.00 ng mL^-1^ (normal range, 0.00-0.07 ng mL^-1^). The serum CK and AST activity ranged from 1654 to 20521 (normal range, 264-562) and 261.00-9141.00 IU L^-1^ (normal range, 84.00-163.00) respectively. Two lambs died and 3 were euthanized due to poor prognosis. At necropsy, the myocardiums were soft and flaccid, showing widespread pale areas in the subepicardial and subendocardial muscles. The size and shape of those lesions was variable and ranged from 3 to 23mm in diameter (Fig. 2). Tissue samples taken from heart were fixed in 10% buffered formalin and processed for routine histopathology. Sections were stained by Hematoxylin and Eosin (H & E).

**Fig. 3:**
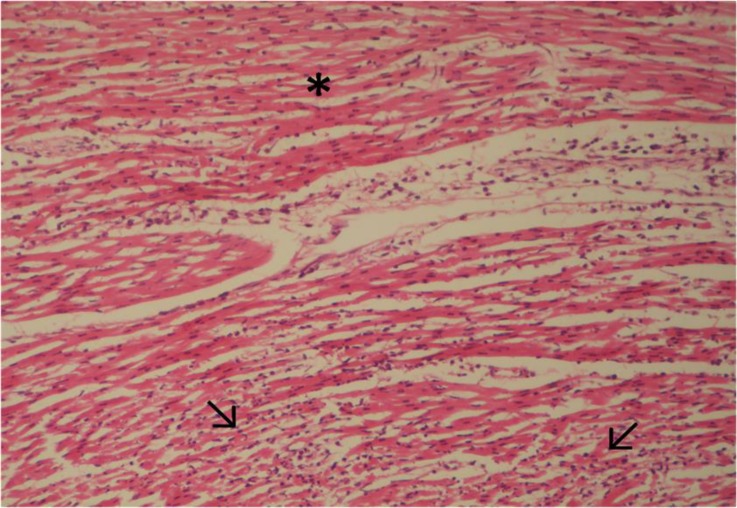
Hyaline degeneration (asterisk) and coagulative necrosis (arrows) of cardiac myocytes. H & E, 320×.

**Fig. 4:**
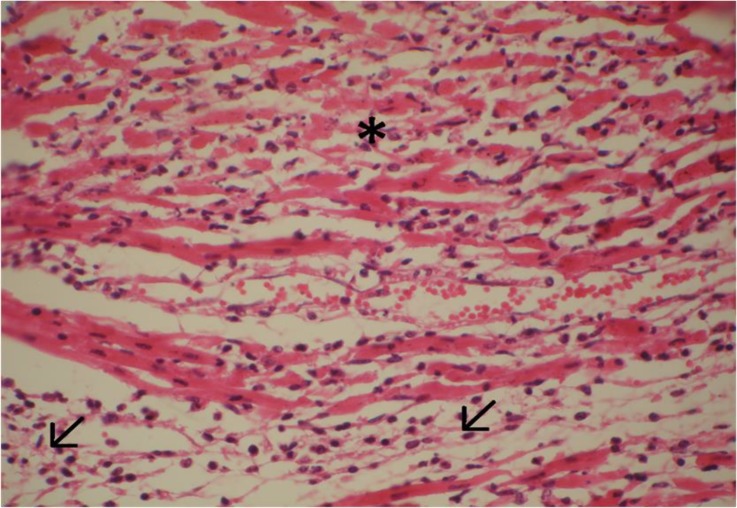
Coagulative necrosis and fragmentation of cardiac myocytes (asterisk) associated with inflammatory infiltration in interstitial space (arrows), H & E, 640×.

Microscopic examination of heart tissues revealed inflammatory infiltration, hyaline degeneration, coagulative necrosis of myocytes with pyknotic nuclei and eosinophilic cytoplasm, and occasionally fragmentation of myocytes. Myocardial necrosis varied from multifocal to confluent in all lambs. Cellular infiltration was composed of neutrophils and mononuclear cells (Figs. 3 and 4).

## Discussion

FMD in young lambs and kids is usually characterized by high mortality without appearance of classic signs of the disease, vesicular and ulcerative lesions in the oral cavity. High mortality due to FMD in young animals is associated to acute myocarditis.^5^^,^^7^^,^^8^ In some epidemics, affected flocks may lose up to 90% of the lamb crop due to cardiac failure.^8^ Clinical signs of such cases including collapse, fever, tachycardia and abdominal respiration,^9^ are not diagnostic for FMD. As indicated in the present report, clinically recognizable arrhythmia, particularly tachyarrhythmias associated with multiple ventricular ectopics accompanied by high levels of serum cardiac troponin I and serum CK and AST activity are features of fatal form of FMD in lambs. The serum concentration of cardiac troponin I is an excellent cardiac biomarker in large animals, providing a sensitive and persistent indicator of myocardial injury.^2^ It has been reported that serum cardiac troponin concentration is an earlier marker of myocardial damage after virus infection than was the histologic finding of inflammation.^14^ Following myocardial damage in humans, cardiac troponins leaks rapidly from myocytes and appears in blood after 2 to 4 hr and persists up to10 to 21 days.^15^ It has also been shown that cardiac troponin I level in serum of sheep increase 1 day after myocardial injury and reduces to physiological levels in next 14 days.^10^ Serum CK and AST activity can be used as indicator of cardiac damage, but they are not specific and can also increase following skeletal muscle damage. Serum AST activities is also elevated during hepatic damage.^11^ However, elevation of serum CK and AST activities in parallel with high levels of serum troponin I can supported the myocardial injury caused by FMDV in lambs. These biochemical findings were also supported by pathological findings of myocardial cell degeneration and necrosis. Similar results have been reported in calves with FMD.^12^^,^^13^

To the authors knowledge the ECG feature of cases affected with FMD has not been reported. Focal or multifocal ventricular premature beats characterized by abnormal amplitude and duration of ORS complexes and T waves are indicative of myocardial diseases.^2^ Progression of ventricular arrhythmias leads to ventricular fibrillation and death of affected animal. Serum troponin I along with ECG can be used as a useful prognostic tool as well as a diagnostic yardstick in predicting the likelihood of survival of young ruminants affected with FMD.

## References

[1] Mason PW, Grubman MJ, Baxt B (2003). Molecular basis of pathogenesis of FMDV. Virus Res.

[2] Radostits OM, Gay CC, Hinchcliff KW (2007). Veterinary Medicine - A textbook of the diseases of cattle, horses, sheep, pigs and goats.

[3] Mann JA, Sellers RF, Dinter Z, Morein B Foot-and-mouth disease virus. Virus infections of vertebrates.

[4] Murphy FA, Gibbs EPJ, Horzinek MC (1999). Veterinary Virology.

[5] Alexandersen S, Zhang Z, Donaldson AI (2003). The pathogenesis and diagnosis of foot-and- mouth disease. J Comp Pathol.

[6] Grubman MJ, Baxt B (2004). Foot-and-mouth disease. Clin Microbiol.

[7] Glubahar MY, Davis WC, Gunenc T (2007). Myocarditis associated with foot-and-mouth disease virus type O in lambs. Vet Pathol.

[8] Kitching RP, Hughes GJ (2002). Clinical variation in foot and mouth disease: sheep and goats. Rev Sci Tech Off Int Epiz.

[9] Pay TWF (1988). FMD in sheep and goats: A review. FMD Bulletin.

[10] Leonardi F, Passeri B, Fusari A (2008). Cardiac troponin I (cTnI) concentration in an ovine model of myocardial ischemia. Res Vet Sci.

[11] Cardinet GH, Kaneko JJ, Harvey JW, Bruss ML (1997). Skeletal muscle function. Clinical biochemistry of domestic animals.

[12] Tunca R, Sozmen M, Erdogan H (2008). Determination of cardiac troponin I in the blood and heart of calves with foot-and-mouth disease. J Vet Diagn Invest.

[13] Karapinar T, Dabak DO, Kuloglu T (2010). High cardiac troponin I plasma concentration in a calf with myocarditis. Can Vet J.

[14] Lim BK, Shin JO, Choe SC (2005). et al. Myocardial injuries occurs earlier than myocardial inflammation in acute experimental viral myocarditis. Exp Mol Med.

[15] Korff S, Katus HA, Giannitsis E (2006). Differential diagnosis of elevated troponins. Heart.

